# DONALD P. KENT AND ROBERT W. KLEEMEIER AWARD LECTURES

**DOI:** 10.1093/geroni/igad104.1492

**Published:** 2023-12-21

**Authors:** James Nelson

## Abstract

The Donald P. Kent Award lecture will feature an address by the 2022 Kent Award recipient Nancy R. Hooyman, PhD, FGSA, of the University of Washington. The Kent Award is given annually to a member of The Gerontological Society of America who best exemplifies the highest standards of professional leadership in gerontology through teaching, service, and interpretation of gerontology to the larger society. The Robert W. Kleemeier Award lecture will feature an address by the 2022 Kleemeier Award recipient Sheryl Zimmerman, PhD, FGSA, of the University of North Carolina at Chapel Hill. The Kleemeier Award is given annually to a member of The Gerontological Society of America in recognition for outstanding research in the field of gerontology.

